# Inter-subject Functional Correlation Reveal a Hierarchical Organization of Extrinsic and Intrinsic Systems in the Brain

**DOI:** 10.1038/s41598-017-11324-8

**Published:** 2017-09-07

**Authors:** Yudan Ren, Vinh Thai Nguyen, Lei Guo, Christine Cong Guo

**Affiliations:** 1 School of Automation, Northwestern Polytechnical University, Xi’an, China; 2 QIMR Berghofer Medical Research Institute, Brisbane, Australia

## Abstract

The brain is constantly monitoring and integrating both cues from the external world and signals generated intrinsically. These extrinsically and intrinsically-driven neural processes are thought to engage anatomically distinct regions, which are thought to constitute the extrinsic and intrinsic systems of the brain. While the specialization of extrinsic and intrinsic system is evident in primary and secondary sensory cortices, a systematic mapping of the whole brain remains elusive. Here, we characterized the extrinsic and intrinsic functional activities in the brain during naturalistic movie-viewing. Using a novel inter-subject functional correlation (ISFC) analysis, we found that the strength of ISFC shifts along the hierarchical organization of the brain. Primary sensory cortices appear to have strong inter-subject functional correlation, consistent with their role in processing exogenous information, while heteromodal regions that attend to endogenous processes have low inter-subject functional correlation. Those brain systems with higher intrinsic tendency show greater inter-individual variability, likely reflecting the aspects of brain connectivity architecture unique to individuals. Our study presents a novel framework for dissecting extrinsically- and intrinsically-driven processes, as well as examining individual differences in brain function during naturalistic stimulation.

## Introduction

Psychologists and philosophers have long postulated that exogenous and endogenous processes are supported by different brain systems, namely the extrinsic and intrinsic systems, respectively^1–4^. On one hand, primary sensory regions, such as calcarine sulcus and Heschl’s gyrus, can be explicitly activated by external sensory stimuli and thus constitute part of the extrinsic system. On the other hand, heteromodal regions that carry out multimodal integration likely belong to the intrinsic system^5^. While the extrinsic system has been well characterized in neuroimaging literature, the intrinsic system is much harder to engage experimentally. Even with tasks specifically designed to elicit endogenous processes^6, 7^, the responses of the intrinsic system were more variable and noisy than the extrinsic system at a group level. A detailed characterization of the extrinsic-intrinsic divisions thus remains a fundamental question in human brain mapping.

While cognitive neuroscience traditionally employed tasks in neuroimaging paradigms, a growing literature now focuses on resting state paradigms, a task-free condition that minimizes extrinsically-driven processes^8, 9^. A major discovery from the resting state literature is the characterization of large-scale, distributed connectivity networks in the absence of external stimuli^10, 11^. These resting state networks were regarded as an intrinsic or endogenous system of the brain, referred to as the default mode network^12–16^. It was soon recognized that high resting state connectivity did not necessarily imply trait preference to endogenous process, as regions in the extrinsic system, such as the primary and secondary visual cortices, also form connectivity networks at rest^17^.

Furthermore, most connectivity networks characterized at resting state actually resemble co-activation patterns during various tasks^18, 19^, as well as functional connectivity networks during these task-evoked conditions^20^. Therefore, resting state connectivity does not provide an indication of intrinsic-extrinsic tendency.

Recently, naturalistic paradigms were used to map the extrinsic and intrinsic brain systems under conditions approximating real-life situations^21, 22^. It is well established that naturalistic paradigms, such as natural movie viewing or story listening, could evoke highly consistent neural responses as measured by inter-subject correlation (ISC) – the correlation of BOLD signals between subjects^23, 24^. The level of consistency is much higher at the primary sensory regions than heteromodal brain regions such as anterior cingulate and insula cortices. Therefore, ISC appears to reflect the tendency of a brain region to be driven by external stimuli. This property has been used to define the intrinsic and extrinsic systems in the posterior region of the brain – regions with significant ISC were regarded as extrinsic and regions with insignificant ISC as intrinsic^21^. Reactivity to external stimuli, however, does not necessarily reflect an involvement with endogenous processes. In addition, this binary division based on a statistical threshold might oversimplify the organization of intrinsic and extrinsic systems in the brain. A quantitative metric that could reflect both extrinsically- and intrinsically-oriented processes is thus needed.

This current study assessed functional connectivity driven by extrinsically- versus intrinsically-oriented activities during a natural viewing paradigm. We used a data-driven approach based on a core graph theoretical metric - degree centrality that measures the connectedness of each brain region - to quantify the extrinsically-driven connectivity of each brain region relative to its intrinsic connectedness. We chose degree centrality because it provide a robust and reliable measure of the global connectivity in the whole brain^25, 26^. Our findings revealed a hierarchical organization of extrinsic-intrinsic tendency in the brain. Furthermore, brain regions with higher intrinsic tendency appear to be more unique to an individual, as measured by inter-subject variability and fingerprint analyses.

## Materials and Methods

### Data acquisition and pre-processing

17 right–handed (10 females, 7males) healthy subjects (ages 27 ± 2.7) participated in the study. The participants were recruited from the University of Queensland and compensated for their participation. Every participant signed a written informed consent. The study was approved by the human ethics committee of the University of Queensland and was conducted according to National Health and Medical Research Council guidelines. The experiment comprised two scanning sessions with an interval of around 3 months. For each session, participants underwent an 8-min resting state fMRI exam with eyes closed, and then freely viewed a 20-min movie *The Butterfly Circus*. This is a short, positively valenced movie that depicts the story of a man born without limbs who is encouraged by the showman of a renowned circus to discover his own potential. All participants reported that they had not previously seen the movie. After completing each scanning session, participants were asked to complete a questionnaire and rate their experience when watching the movie, including the level of boredom, enjoyment, valence, as well as the audio and video quality of the movie during fMRI acquisition, on the scale between 1 and 5 (Table 1). Note that all participants reported positive viewing experience afterwards (Table 1). The movie stimulus was presented using the Presentation software (NeuroBehavioral Systems, USA) and displayed via an MRI-compatible monitor located at the rear of the scanner. The soundtrack of the movie was delivered through MRI-compatible audio headphones (Nordic NeuroLab, Norway). After 3 months, all the subjects were scanned with the same protocol (session B).

**Table 1:** Affective ratings of the movie under session A and session B (mean ± standard deviation).

Session	Boredom	Enjoyment	Valence	Audio/video quality
A	1.48 ± 0.81	4.05 ± 1.07	3.62 ± 1.07	3.95 ± 0.86
B	2.43 ± 1.03	3.33 ± 1.11	3.43 ± 0.60	4.14 ± 0.79

All structural and functional images were acquired from a whole-body 3 T Siemens Trio MRI Scanner. A high-resolution T1-wieghted MPRAGE structural image was acquired for each subject with following parameters: TR = 4000 ms, TE = 2.89 ms, FA = 9°, FOV = 240 mm × 256 mm, and voxel resolution 1mm × 1 mm × 1 mm. The scanning parameters for the functional scan were: TR = 2200 ms, TE = 30 ms, FA = 79°, FOV = 192 mm × 192 mm, voxel resolution 3 mm × 3 mm × 3 mm, and 44 slices with in-plane resolution of 64 × 64. Functional images were preprocessed using Statistical Parametric Mapping toolbox (SPM12). The preprocessing pipeline included slice timing correction and realignment using a six-parameter linear transformation, co-registration, normalization, spatial smoothing with 6 mm full width half maximum Gaussian kernel, and band pass filtering (0.0085–0.15 Hz). After band pass filtering, nuisance covariates including WM, CSF and motion parameters were then regressed out using the Data Processing Assistant for Resting-state fMRI software (DPARSF) to reduce potential effects of physiological confounds^27, 28^.

### With-subject functional connectivity analyses

Both within-subject functional connectivity and inter-subject functional correlation analyses were performed on established parcellation atlases that cover the whole brain. Two atlases were used: the 200 ROI atlas based on Craddock 2012 parcellations^29^ and the 513 ROI atlas based on a random parcellation algorithm^30^. Cerebellar ROIs in the Craddock atlas were excluded in the current analysis, resulting in 181 ROIs. The 513 ROI atlas does not include the cerebellum. Analyses using the two atlases yielded similar results. We thus presented results based on the Craddock atlas in the main text, and results based on the 513 ROI atlas in the Supplementary Materials as a further validation (Supplementary Figs S1–S3). Note that the brains in the figures were visualized using a brain network visualization tool, BrainNet Viewer^31^.

Time series of ROIs were first extracted from preprocessed fMRI data. Pearson correlation was calculated between each pair of ROIs’ time series separately for each condition and each session, resulting in a functional connectivity matrix for each subject under each condition and each session. Then, the correlation coefficients were transformed to z-scores using Fisher’s transformation, averaged across all subjects for each condition, and then reverted to Pearson’s r values to derive group-level functional connectivity matrix^32^.

### Inter-subject functional and inter-subject correlation analyses

**Figure 1:**
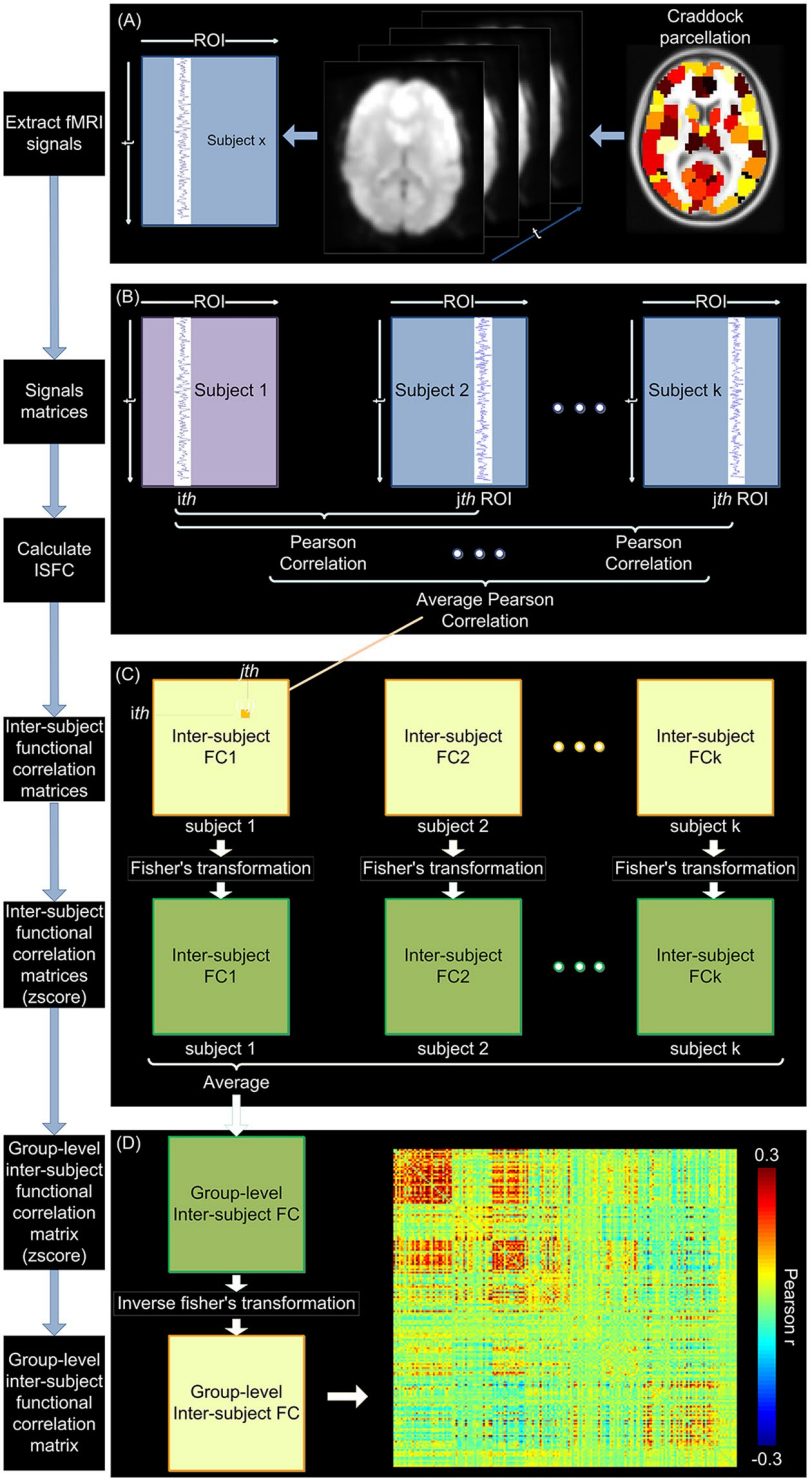
Schematic of the inter-subject functional correlation method^32^. Color bar in (D) signifies Pearson’s correlation coefficients between each pair of ROIs.

To calculate inter-subject functional correlation (ISFC), ROIs’ time series were extracted from preprocessed naturalistic fMRI data under each session (Fig. 1A). Rather than computing the correlations across brain regions within subject, ISFC were calculated across brain regions across subjects^32^. For each subject in the group, Pearson correlations were first calculated between each ROI’s time series of this subject and any other ROI’s time series of another subject, generating one functional connectivity matrix between these two subjects (Fig. 1B). This calculation was then repeated between this subject and every other subject in the group, resulting in 16 connectivity matrices. These matrices were then averaged to generate one ISFC matrix for each subject (Fig. 1C). For group-level ISFC matrix, the correlation coefficients were transformed to z-scores using Fisher’s transformation, averaged across all subjects, and finally reverted to Pearson’s r values (Fig. 1C and D)^32^.

Inter-subject correlation (ISC) within a group was calculated as an average correlation $R=(1/N){\sum }_{j=1}^{N}{r}_{j}$ at each ROI, where r_j_ is the mean of Pearson correlations between that ROI’s time series in one subject and the that ROI’s time series in each of the remaining subjects in the group^23, 24, 33^.

### Graph theory analyses on ROI connectivity matrices

We used a basic graph theoretical metric, degree centrality, to quantify network connectivity properties for both within-subject functional connectivity and inter-subject functional correlation matrices^34^. Connectivity matrices were first thresholded to determine the presence or absence of connections between ROIs. As there is no standard thresholding approach for graph theoretical analysis, we repeated all our analyses using several levels of thresholds based on either correlation strength or sparsity.

The main analyses were performed using thresholds based on correlation strength. Since within-subject functional connectivity and inter-subject functional correlation has qualitatively different values, to allow for statistical comparisons (see below), each connectivity matrices were normalized by subtracting the mean value and then dividing by the standard deviation. Weighted degree centrality were from the normalized adjacency matrices using Brain Connectivity Toolbox^34^, as the weighted sum of all the supra-threshold edges connected to the nodes. Here, to ensure robustness of our results to the level of threshold, we repeated our analyses with three different thresholds of 0.1, 0.3 and 0.5 (Supplementary Fig. S4).

To furthermore ensure the robustness of our results, we repeated our analyses using thresholds based on sparsity. Here a common level of network sparsity was applied to each connectivity matrices (normalisation is not needed) and degree centrality was calculated as the number of suprathreshold edges connected to the node^34^. Again, we repeated our analyses with three different sparsity thresholds of 10%, 30% and 50% (Supplementary Figs S5–S8).

Similar approaches were used to calculate degree centrality of the inter-subject functional correlation matrices. Note that inter-subject functional correlation matrices were asymmetric: the element (i,j) represented the correlation between the i^th^ ROI’ time series of one subject and j^th^ ROI’s time series of the remaining subjects, while the the element (j,i) represented the correlation between the j^th^ ROI’ time series of one subject and i^th^ ROI’s time series of the remaining subjects (Fig. 1). Thus, we calculated directed degree centrality on adjacency matrices of inter-subject functional correlation, as the average sum of both inward and outward edges connected to the node.

### Statistical analyses and divisions of extrinsic-intrinsic systems

To assess the differences between within- and inter-subject degree centrality, we performed paired t-tests between within- and inter-subject degree centrality for each ROI across all subjects. T values derived from this paired t-test were defined as the IE (intrinsic/extrinsic) index for each brain region. We then divided all brain regions into five equal portions according to their IE indices, resulting in a continuum of brain systems along the axis of extrinsic-intrinsic tendency.

To compare functional connectivity measures between natural viewing and resting-state, we derived functional connectivity matrices and degree centrality using the last 8 min segment of natural viewing fMRI data, matched with the length of resting state fMRI data. Then we performed paired t-test on both connectivity matrices and degree centrality maps between these two conditions. Note that all the statistical comparison results in our study were thresholded using an FDR-corrected p < 0.05.

### Inter-subject variability analyses

To estimate inter-subject variability^35, 36^, first functional connectivity maps were calculated by taking each of the 181 ROIs’s time series as seed and correlating with the remaining ROIs’ time series, resulting in 181 functional connectivity maps for each subject. We defined the connectivity map based on each ROI as Fi(s), where *i* = 1,2,…181, and *Fi* is a 1 × 181 vector, s represents the subject.

For a given ROI *i*, the similarity between the 17 maps derived from 17 subjects was computed by averaging the correlation values between any two maps:

$${R}_{i}=E[corr({F}_{i}({s}_{p}),{F}_{i}({s}_{q}))],$$

where

1 $$p,q=1,2,\mathrm{...17};p\ne q.$$

The inter-subject variability for each ROI was defined as following:

2 $${V}_{i}=1-{R}_{i}$$

The inter-subject variability was calculated under resting-state and natural viewing respectively.

### Functional connectivity fingerprinting analyses

A functional connectivity fingerprint method was used to assess the accuracy of individual identification^37^. First, within-subject functional connectivity matrices were calculated for each subject under each condition, resulting in 4 matrices (two matrices for resting-state, two matrices for natural viewing). Individual identification was conducted between each pairs of scans, where one is used as the ‘target’ session and the other as the ‘database’ session. In each iteration, one subject’s connectivity matrix from the target set was selected and compared against each of connectivity matrices in the database set to determine the matrix of maximum similarity. Similarity was defined as the Pearson correlation coefficient between two vectors of edges values taken from the target matrix and each of the database matrices. The matrix with maximum similarity is then identified as the target identity. If this predicted identity matched the true identity, the iteration was assigned a score of 1, and 0 if it did not. For each target-database pair, the connectivity matrix of every subject in target set was compared against the matrices in database set once. The identification rate was defined as the percentage of iterations where the identity was correctly predicted out of the total number of iterations. In our study, we tested the identification between two resting-state datasets and between two natural viewing datasets from two scan sessions separately.

We also derived identification confidence to evaluate the performance of fingerprint individual identification. In each iteration, identification confidence was based on the differences between the highest similarity and the average of the remaining similarity, where identification confidence was assigned as positive if the predicted identity matched the true identity, and negative if it did not. For each target-database pair, the identification confidence was averaged across all the iterations.

We computed identification accuracy and confidence using ROIs from the whole brain as well as ROIs from each of the five extrinsic-intrinsic systems. In the latter case, only the connections between ROIs within each system were included in the identification tests.

### Data availability

The datasets generated and analyzed during this study are available from the corresponding author on reasonable request.

## Results

### Network analysis of inter-subject functional correlation

**Figure 2:**
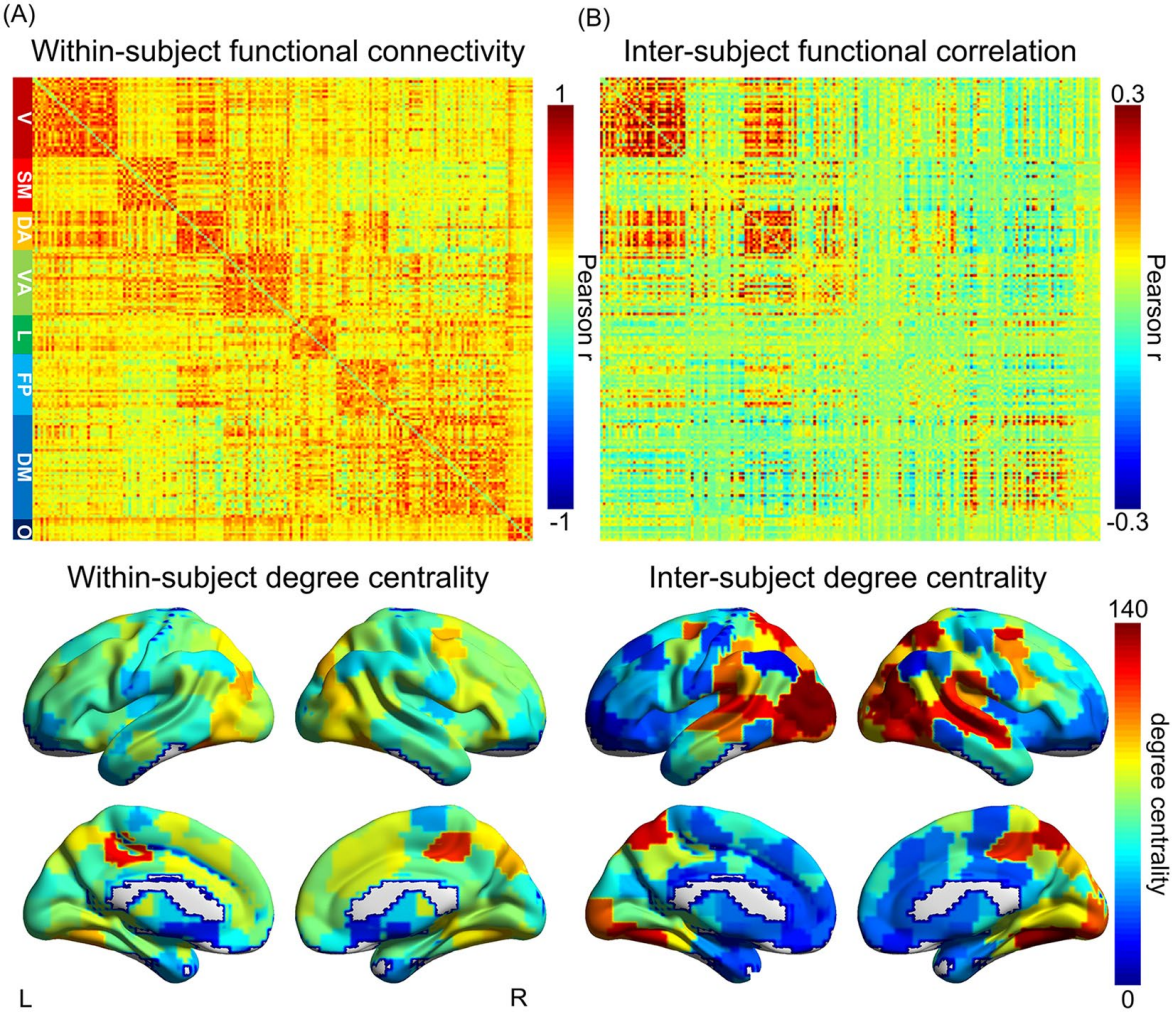
Within-subject functional connectivity and inter- subject functional correlation matrix and degree centrality map. (**A**) Group-level within-subject functional connectivity matrix (upper panel) and degree centrality map (lower panel) during natural viewing; (**B**) Group-level inter-subject functional correlation matrix (upper panel) and degree centrality map (lower panel) during natural viewing (T = 0.1). ROIs are organized according to the 7-network scheme 11 as labeled on the left of panel (A) (V: visual, SM: Somatomotor, DA: Dorsal attention, VA: Ventral attention, L: limbic, FP: Frontoparietal, DM: Default mode, O: Other regions). Color bars on the upper panel signify Pearson’s correlation coefficients between each pair of ROIs. Color bar on the lower panel signifies degree centrality.

Large-scale network organization is fundamental to human brain function. Robust and reliable connectivity architecture can be observed from functional connectivity matrices during both resting state and natural viewing conditions^38–40^ (Fig. 2A, Supplementary Fig. S9). We here adopted a novel approach to assess the network characteristics that are primarily influenced by external stimuli (See Methods for detail). This approach first computed inter-subject functional correlation (ISFC) – high scores indicate connections that are consistent across subjects and hence time-locked to the external stimuli^32^. We then derived an inter-subject degree centrality for each brain region from these ISFC matrices. As expected, the distribution of inter-subject degree centrality similarly resembles that of ISC map, that is, brain regions with high ISC tend to show a high level of inter-subject degree centrality (Fig. 2B, Supplementary Fig. S10).

On the other hand, the ISFC matrix and degree map also show similar patterns as the ones derived from within-subject functional connectivity, albeit with weaker correlation strength (Fig. 2, upper panel). In addition, hub regions such as the precuneus showed higher inter-subject degree centrality than other nodes of comparable ISC (Fig. 2B, Supplementary Fig. S10)^41^. Therefore, inter-subject degree centrality likely reflects the combined effects from the externally-driven processes and the inherent functional connectivity network topology. We thus hypothesized that the divergence between inter- and within-subject degree centrality could serve as an index of the relative tendency to extrinsic versus intrinsic processes for each brain region.

To avoid potential bias introduced by the choice of thresholds and ROI atlas, we repeated our analyses using several levels of thresholds based on either correlation strength or sparsity, and two parcellation atlas (See Methods for details). The findings are consistent across these analytical choices, hence we presented the main results using the absolute threshold of 0.1 and the Craddock ROIs atlas. Additional results were included in the Supplementary Materials.

### Contrast between within- and inter- subject degree centrality

**Figure 3:**
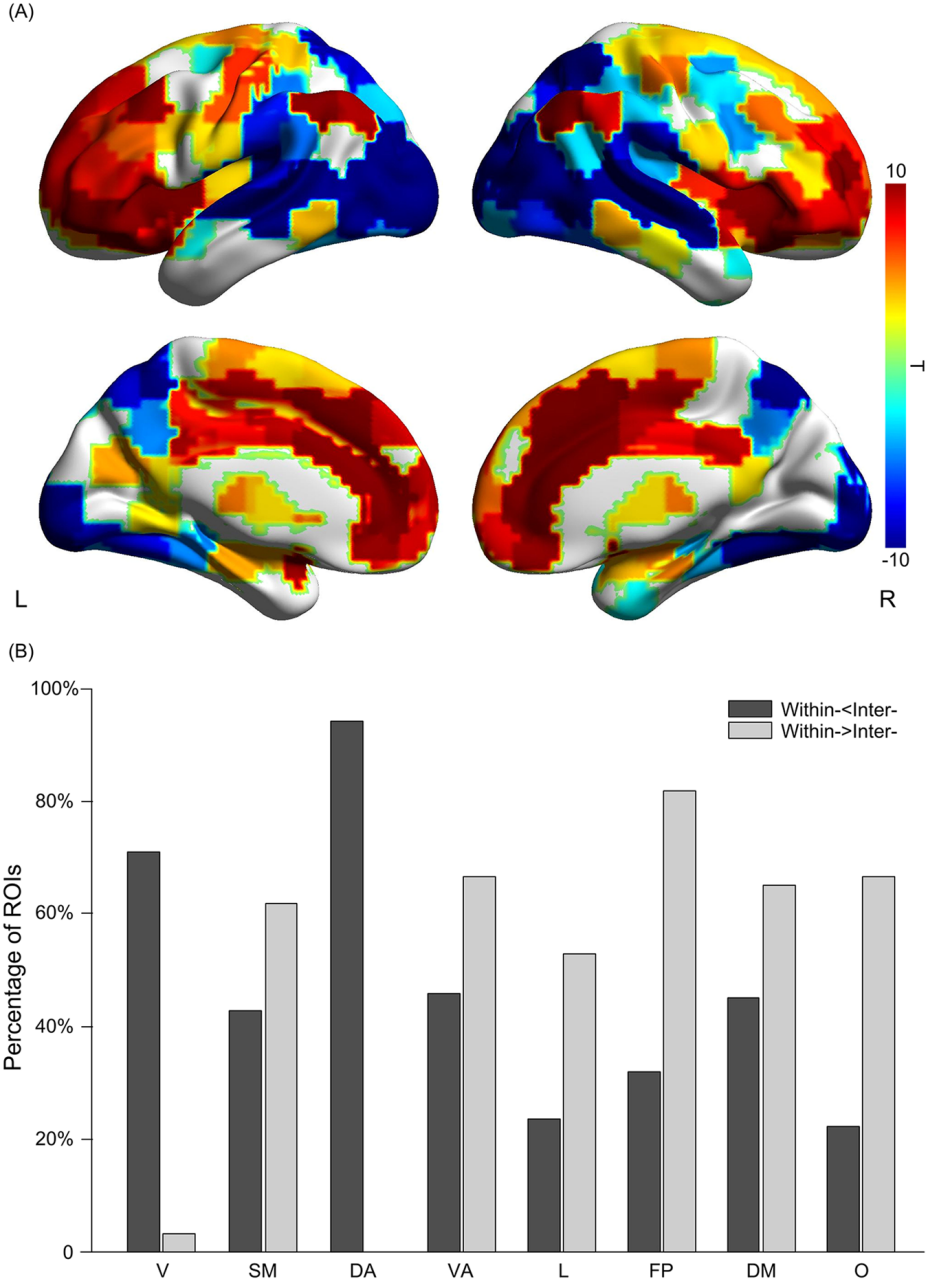
Contrast between within- and inter- degree centrality. (**A**) Statistical comparison between within- and inter-subject degree centrality during natural viewing. This statistical comparison indicates a posterior to anterior gradient in the brain. Color bar signifies the T statistics (warm color, within- > inter-; cool color, within- < inter; paired t-test, FDR-corrected p < 0.05). (**B**) The percentage of ROIs in each network showing significant differences between within- and inter-subject degree centrality, which reveals that many networks are composed of both extrinsic and intrinsic nodes. ROIs are sorted into the 7-network scheme 11 as in Fig. 2. Dark gray, within- < inter-; Light gray, within- > inter-.

We derived the statistical differences between inter- and within-subject degree centrality for the whole brain. This comparison revealed a posterior to anterior gradient in the brain: significantly higher level of inter-subject degree centrality is observed in occipital, parietal, temporal and posterior insular cortices, whereas significantly higher within-subject degree centrality is found in the prefrontal regions, anterior insula and cingulate cortices (Fig. 3A; paired t-test, FRD-corrected p < 0.05). In addition to this posterior to anterior gradient, we also noticed a lateral to medial gradient where the medial wall is predominantly occupied by intrinsic systems (Fig. 3A, paired t-test, FDR-corrected p < 0.05). This pattern is consistent with known functional neuroanatomy – temporal and occipital lobes are selective for audiovisual processing and prefrontal regions for higher-order cognition – supporting the use of this comparison between inter- and within-subject degree centrality to index the extrinsic-intrinsic tendency.

It is often assumed that brain regions within the same functional networks share similar extrinsic-intrinsic tendency – the default mode network (DMN), encompassing the precuneus, posterior cingulate cortex, vmPFC and bilateral angular gyrus, is referred to as an intrinsic network all together^12–16^. Our results, however, suggest many networks are composed of both extrinsic and intrinsic nodes (Fig. 3B). Within the DMN, while the bilateral angular gyrus and vmPFC are intrinsic, the precuneus shows robust extrinsic tendency (Fig. 3A). Similarly, the ventral attention and somatosensory networks also contain almost equal proportion of extrinsic and intrinsic nodes (Fig. 3B). Only visual and dorsal attention networks appear to be predominantly extrinsic. Overall, most resting state networks, despite of high intra-network connectivity (Fig. 2A), contain a mixture of extrinsic and intrinsic nodes and thus appear to engage in both exogenous and endogenous processes (Fig. 3).

### Divisions of extrinsic and intrinsic systems during natural viewing

**Figure 4:**
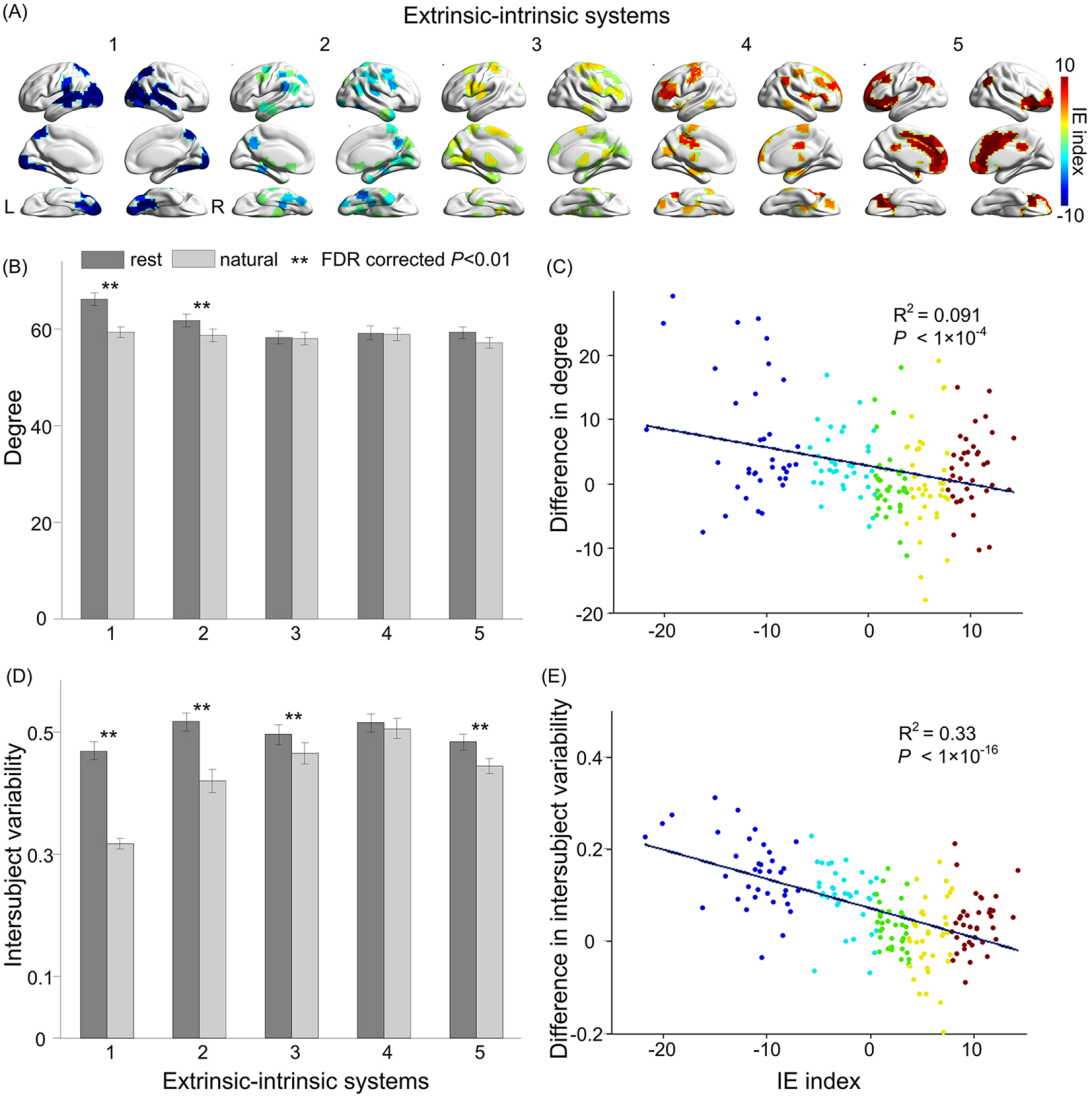
Hierarchical organization of extrinsic-intrinsic systems. (**A**) Division of all ROIs into five extrinsic-intrinsic systems based on the IE index. Color bar signifies the IE index (warm color, intrinsic systems; cool color, extrinsic systems). (**B**) Average degree centrality of the five extrinsic-intrinsic systems during resting state and natural viewing (**represents significant difference between resting state and natural viewing; paired t-test, FDR-corrected p < 0.01). Error bars signify standard error of the mean. (**C**) Correlation between the IE index and changes in degree centrality between resting state and natural viewing (rest – natural). ROIs are color-coded according to the extrinsic-intrinsic division. (**D**) Average inter-subject variability of the five extrinsic-intrinsic systems during resting state and natural viewing (**represents significant difference between resting state and natural viewing; paired t-test, FDR-corrected p < 0.01). Error bars signify standard error of the mean. (**E**) Correlation between the IE index and changes in inter-subject variability between resting state and natural viewing (rest – natural). ROIs are color-coded according to the extrinsic-intrinsic division.

Our analysis further suggests that the extrinsic-intrinsic tendency appears to distribute along a continuous spectrum, rather than a binary division. To assess this continuous distribution in detail, we used the statistical difference scores, referred to as the intrinsic-extrinsic (IE) indices, to divide all brain regions into five equal portions according to their IE indices (Fig. 4A). Consistent with our previous observations, the posterior midline DMN nodes belong to the two most extrinsic divisions, in contrast to the other DMN nodes being mostly intrinsic. The definition of the IE index and divisions hence allowed us to further investigate the engagement of extrinsic and intrinsic systems during different behavioral conditions.

Since some of the intrinsic nodes, such as the angular gyrus and anterior insula have been previously characterized as the hub regions in the brain^42, 43^, we asked whether intrinsic systems are more connected in general. Degree centrality, however, did not differ significantly across intrinsic and extrinsic systems during natural viewing (Fig. 4B). During resting state, there is a trend that the extrinsic systems are associated with higher degree centrality (Fig. 4B). This observation again suggested that the extrinsic-intrinsic tendency is relatively independent from the topology of functional connectivity in the brain.

Finally, we assessed the changes in functional connectivity between resting and movie viewing conditions across the identified five extrinsic-intrinsic systems. Significant differences were observed only for the two extrinsic systems, whose degree centrality was lower during movie viewing than resting state (Fig. 4B; paired t-test, FRD-corrected p < 0.01). The intrinsic systems, however, did not show changes in their connectivity between behavioral conditions. Overall, there was a weak correlation between with the IE index and changes in degree centrality between conditions, mostly driven by the changes in the extrinsic systems (Fig. 4C).

### Intrinsic systems have high inter-subject variability

While the extrinsic and intrinsic systems did not differ much in the absolute level of degree centrality, systematic differences were found with the inter-subject variability of degree centrality. Inter-subject variability is significantly reduced during natural viewing compared to resting state (Fig. 4D; paired t-test, FDR-corrected p < 0.01). Furthermore, these changes in variability significantly correlated with the IE index, with the extrinsic systems showing the greatest decreases in variability during natural viewing (Fig. 4E; R^2^ = 0.33, p < 1 × 10^−16^). The low variability of extrinsic systems during natural viewing is expected given the robust ISC of their neural responses (Supplementary Fig. S10). On the other hand, neural activities of the intrinsic systems, despite the lack of ISC, became less variable during natural viewing. This reduction in variability, although small, suggests that natural viewing condition had an influence on the endogenous processes in the brain^44^ (Fig. 4E).

### Intrinsic systems preferentially represent individuality

**Figure 5:**
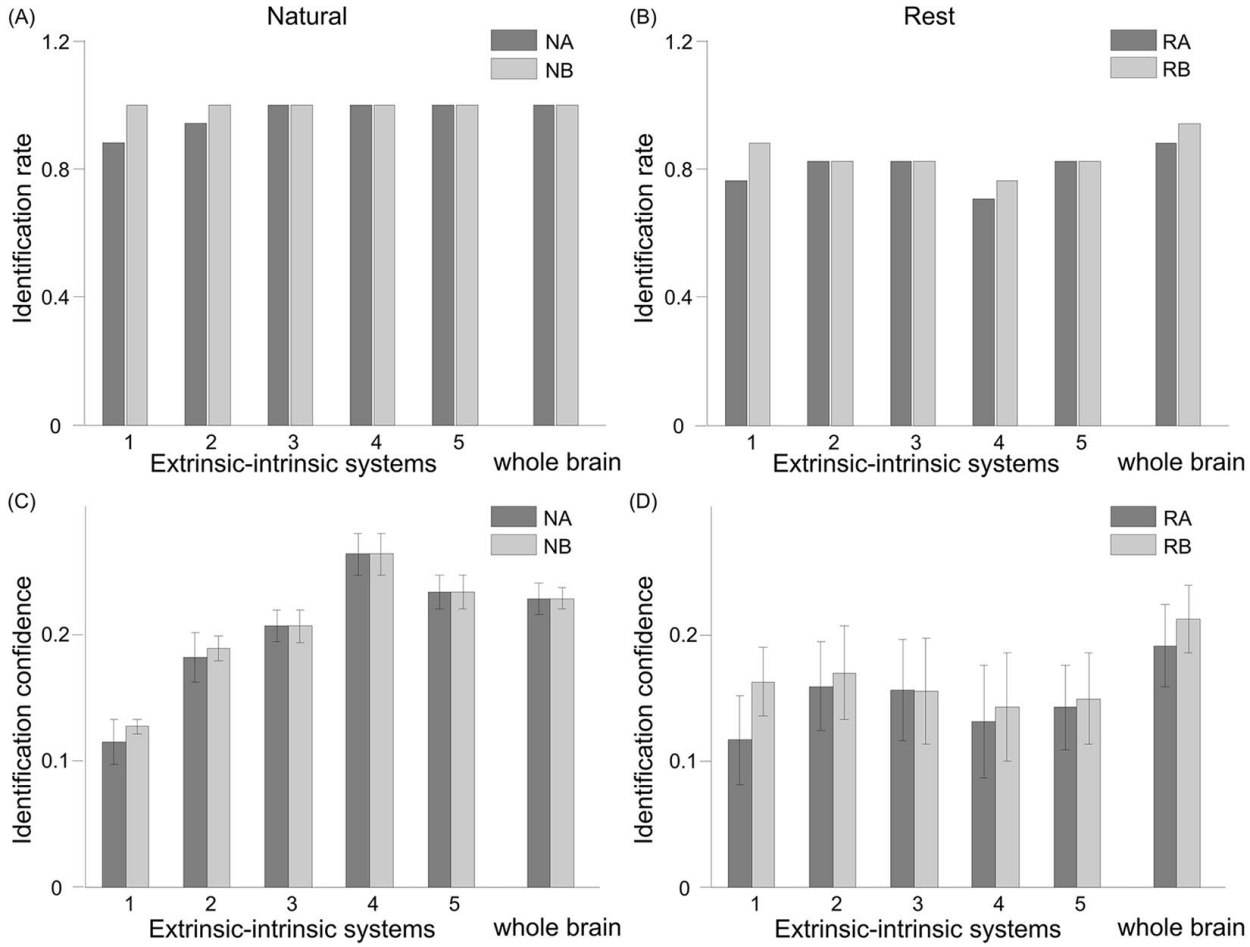
Identification rate and confidence across sessions and extrinsic-intrinsic systems using fingerprint analysis. Bar shading (light or dark gray) indicates the session used as the target (with the other session serving as the database). NA: Natural viewing session **A**; NB: Natural viewing session **B**; RA: Resting state session **A**; RB: Resting state session **B**. Error bars signify standard error of the mean.

The intrinsic systems are engaged by external stimuli, but with lower consistency and higher inter-subject variability than the extrinsic systems (Fig. 4D, Supplementary Fig. S11C). The low consistency was thought to represent noise in their neural responses to external stimuli^32^. Alternatively, high variability could reflect individual differences in the endogenous processes, which depend greatly on prior experience and belief. To test this possibility, we employed the functional connectivity fingerprint analysis that was recently developed for individual identification using resting state functional connectivity^37^. We first validated this method on the resting state data in our dataset, and achieved similar level of identification rates as previously found with the HCP dataset (Fig. 5B, whole brain). When applied to the natural viewing data, this method achieved even higher identification rate than resting state data, reaching to 100% for most tests (Fig. 5A), suggesting that neural responses to natural viewing could be more unique and distinctive than spontaneous neural activity at rest. Then we applied fingerprint analysis on the five systems separately. Intriguingly, the identification confidence is much higher for the intrinsic systems than the extrinsic system during natural viewing (Fig. 5C), suggesting that the high variability in the intrinsic systems is more likely to represent individuality rather than noise. Such pattern was not observed with resting state connectivity (Fig. 5B and D).

## Discussion

Using a novel inter-subject functional correlation approach^32^, we characterized a posterior-to-anterior hierarchical organization of extrinsic and intrinsic systems in the brain. Most regions in the occipital, parietal and temporal cortices respond promptly to external audiovisual stimuli, while prefrontal cortex, anterior insula and cingulate cortices are more likely to represent the derived information of external stimuli that are idiosyncratic and variable across individuals.

Previous studies revealed that the level of ISC reflects the involvement of brain regions with external sensory stimulus, where primary sensory cortices tend to have higher ISC, whereas some higher order brain regions such as anterior cingulate, insula and prefrontal cortices have lower ISC^23, 24^. This coherence effect then naturally divides the cortex into two systems: a system of regions that manifest an across-subject, shared response to external stimulus versus regions that are linked to unique, individualised variations^45^. In our study, ISFC reveals the extent of neural responses driven by external stimulus, while FC primarily reflects the correlation patterns of intrinsic neural signal^32^. Thus, extending upon previous work, we employed the divergence between inter- and within-subject degree centrality as an index of the relative tendency to extrinsic versus intrinsic processes for each brain region.

The organization of extrinsic and intrinsic systems is not confined by the functional connectivity architecture, with most functional connectivity networks comprising both nodes with extrinsic and nodes with intrinsic tendency. Consistent with previous studies, we found neural responses in the intrinsic systems, compared to the extrinsic system, are less consistent during natural viewing. This low level of consistency, however, might not simply reflect neural noise, but rather a high degree of individuality and uniqueness in intrinsically-oriented processes under natural stimulation.

Our approach is designed to characterize the extrinsic and intrinsic tendency during a particular behavioral condition, by comparing inter-subject functional correlation to within-subject functional connectivity derived from this behavioral condition. Alternatively, one could use within-subject connectivity during resting state acquisition to quantify intrinsically-oriented processes. We found this approach – with the 8-min resting state scan acquired in the same session – generated similar extrinsic-intrinsic divisions as our main method (compare Supplementary Fig. S12 with Fig. 3A). This similarity is expected as the general functional connectivity architecture appears to be relatively stable across behavioral states, with some differences in the extrinsic regions (Fig. 4B, Supplementary Fig. S9)^39^. While resting state is arguably a closer approximation of endogenous processes, our method was chosen for three reasons. First, this approach compares the two types of connectome patterns derived from the same data, and thus is not impacted by differences in scan acquisition such as motion and fatigue. Second, this approach does not require an additional resting state acquisition of the same length and thus allows the most efficient use of scan time. Third, resting state acquisition also suffers from drawbacks due to its unconstraint nature and proneness to sleep and motion, resulting in less reliable assessment of connectivity^28, 40^. In addition, resting state is not completely free from external input (acoustic noise). Overall, within-subject connectivity during natural viewing is likely a better measure to match with and control for the inter-subject functional correlation measures.

### Making inference on the internally-driven processes

Unlikely externally-driven processes, which can be generated and manipulated in the laboratory, internal processes cannot be measured and studied directly. Rather, it is often inferred by the inability to be driven directly and reliably by external signals^21, 23, 24^. Here, the BOLD signals are assumed to be the sum of stimulus-induced signals, intrinsic neural signal and non-neuronal noise signal^32, 46–50^. During natural viewing of the same movie stimulus, signals in sensory cortices are highly correlated and “shared” across subjects, reflecting externally-driven processes. On the other hand, higher-order brain regions show “idiosyncratic” signals and are thought to be processing internal information. Previous and our studies thus used this concept to define extrinsic/intrinsic tendency^21, 23, 24^, i.e., through the quantifications of “shared” and “idiosyncratic” signals. This approach certainly has limitations, but offers a useful construct for studying the internal process in human mind, given no direct measurement can be made.

### Hierarchical brain organization

Our results revealed a hierarchical arrangement of extrinsic-intrinsic systems rather than a binary division. Hierarchical processing is a key principle of neural computation in the brain^51–54^. Sensory information of the external world is first represented in the primary sensory cortices, before hierarchical cascading to the secondary sensory cortices and ultimately the higher order cortices. Such hierarchical processing has recently been mathematically formulated under the framework of predictive coding and free energy: each layer of this hierarchical architecture comprises a probabilistic generative model that can make predictions about ascending input and then refine these predictions by minimizing prediction errors^55^. For sensory cortices in the extrinsic systems, inference is made on predictions about the external sensory stimulus, including luminosity, motion and contextual complexity of movie scenes, global attentional impact of scenes, etc^23^, and thus likely to be highly consistent across subjects. For higher order cortices in the intrinsic systems, however, predictive models are generated on ascending inputs after several layers of processing and integration, and are hence influenced by prior experience unique to each individual, resulting in greater variability across subjects. These higher-order regions with higher intrinsic tendency thus manifest endogenously oriented mental processes, such as self-referential processing^56^, problem solving and task-independent thoughts^57^, social cognition^7, 58^.

The hierarchical distribution of inter-subject variability has been previously suggested for functional connectivity at resting state. Resting state connectivity measures show greater variability in heteromodal association cortices than unimodal cortices^35^. The authors proposed that these brain regions with high variability co-localize with brain regions predicting individual differences in cognition and behavior, based on a meta-analysis. Our results here provide direct support of this notion. Using the extrinsic-intrinsic index we developed, we found inter-subject variability was much greater in intrinsic systems than extrinsic systems, especially during natural viewing. Furthermore, higher variability concurs with more accurate identification of an individual from a group (Fig. 5), suggesting neural responses in intrinsic systems are highly variable across subject and yet consistent during two separate viewing sessions. The variable responses of intrinsic systems might provide the neural basis of inter-subject differences in film preferences and other socioemotional behaviors, rather than noise.

### Intrinsic systems and resting state network

When functional connectivity patterns were initially characterized during resting state, they were thought to reflect spontaneous, intrinsic activity^8, 9^. The most studied network, the DMN, is often referred to as an intrinsic network that attends to internal thoughts and mind wandering^12–16^. In contrast, our results further demonstrated that brain nodes within the DMN, despite of high connectivity among themselves, could manifest either extrinsic or intrinsic attributes. The anterior DMN, the VMPFC, and the lateral DMN, the bilateral angular gyrus, are strongly intrinsic (Fig. 3). The precuneus, on the other hand, appears to be extrinsic and shows highly consistent neural responses across subjects. This engagement of the precuneus during natural perception is consistent with previous findings that the precuneus could be activated by a variety of tasks, including visuo-spatial imagery, episodic memory retrieval and theory of mind (for a review see ref. 41). The diverse functional engagement and widespread connectivity of the precuneus could reflect its unique role in integrating both intrinsically and extrinsically driven information within the DMN^59^. Furthermore, the integration of endogenous and exogenous processes seems to be common among functional connectivity networks (Fig. 3), suggesting the functional organization of brain is likely both modular and hierarchical.

### Limitations and future directions

Our study has several limitations. First, we only employed the graph analytical metric, degree centrality, to identify our IE index, as it provide a robust and reliable measure of the whole brain connectivity^25, 26^. Future work could further extend our analysis using other graph metrics, such as ref. 34. Moreover, the individual identification revealed by the fingerprint analyses might not be fully accounted by functional connectivity patterns. Rather, a significant effect might be contributed by inter-subject differences in brain morphology. Nonetheless, we found identification rate and accuracy are higher for natural viewing than resting state conditions, consistent with recent reports^32, 60^ – note this improvement is independent of potential contribution from brain morphology and anatomical connectivity. Another limitation of this analysis is that the identification rate during natural viewing suffers from ceiling effects and could not reveal much difference between intrinsic and extrinsic systems (Fig. 5A)^60^. This ceiling effect might be reduced if a bigger sample is used. Nonetheless, our fingerprint analysis on resting state condition showed very similar results compared to the original analysis on HCP dataset (126 subjects)^37^, suggesting the effects identified in our study are robust and representative. In addition, the interval of two sessions in our datasets was longer than most fingerprint analyses intervals, suggesting the robustness of our results^37, 60^. Finally, as our analysis was performed on one types of behavioral condition, the extrinsic-intrinsic map could change in response to different external stimuli. Further studies could apply our approach to other task paradigms to characterize how endogenous and exogenous processes might be influenced by specific task conditions.

## Electronic supplementary material

supplementary figures
