# Supplementary material for: Inter-subject Functional Correlation Reveal a Hierarchical Organization of Extrinsic and Intrinsic Systems in the Brain
Source: Sci Rep. 2017 Sep 7;7:10876. doi: 10.1038/s41598-017-11324-8 (PMC5589774; doi:10.1038/s41598-017-11324-8)
Supplement: Supplementary file 1 — supplementary figures [file 41598_2017_11324_MOESM1_ESM.pdf]

**Inter-subject Functional Correlation Reveal a Hierarchical Organization of Extrinsic and Intrinsic Systems in the Brain**

Yudan Ren[1,2], Vinh Thai Nguyen[2], Lei Guo[1], Christine Cong Guo[2*]

[1]School of Automation, Northwestern Polytechnical University, Xi'an, China;

[2]QIMR Berghofer Medical Research Institute, Brisbane, Australia.

[*]corresponding authors.

## Supplementary materials

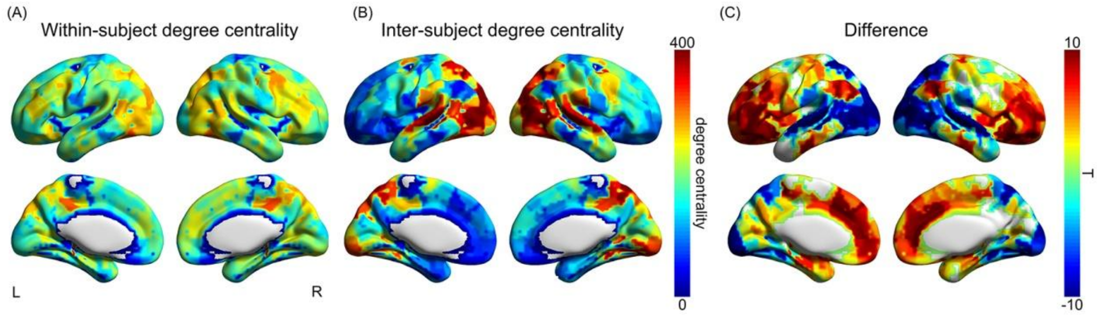


**Fig. S1 Within- and inter- subject degree centrality maps, and statistical comparison between them using 513 ROI atlas.** (A) Group-level within-subject degree centrality map, (B) inter-subject degree centrality map, and (C) statistical comparison between them during natural viewing using 513 ROI atlas. Color bar on the right of the inter-subject degree map signifies degree centrality. Color bar on the right of the difference map signifies the T statistics (warm color, within- > inter-; cool color, within- < inter; paired t-test, FDR-corrected $p < 0.05$).

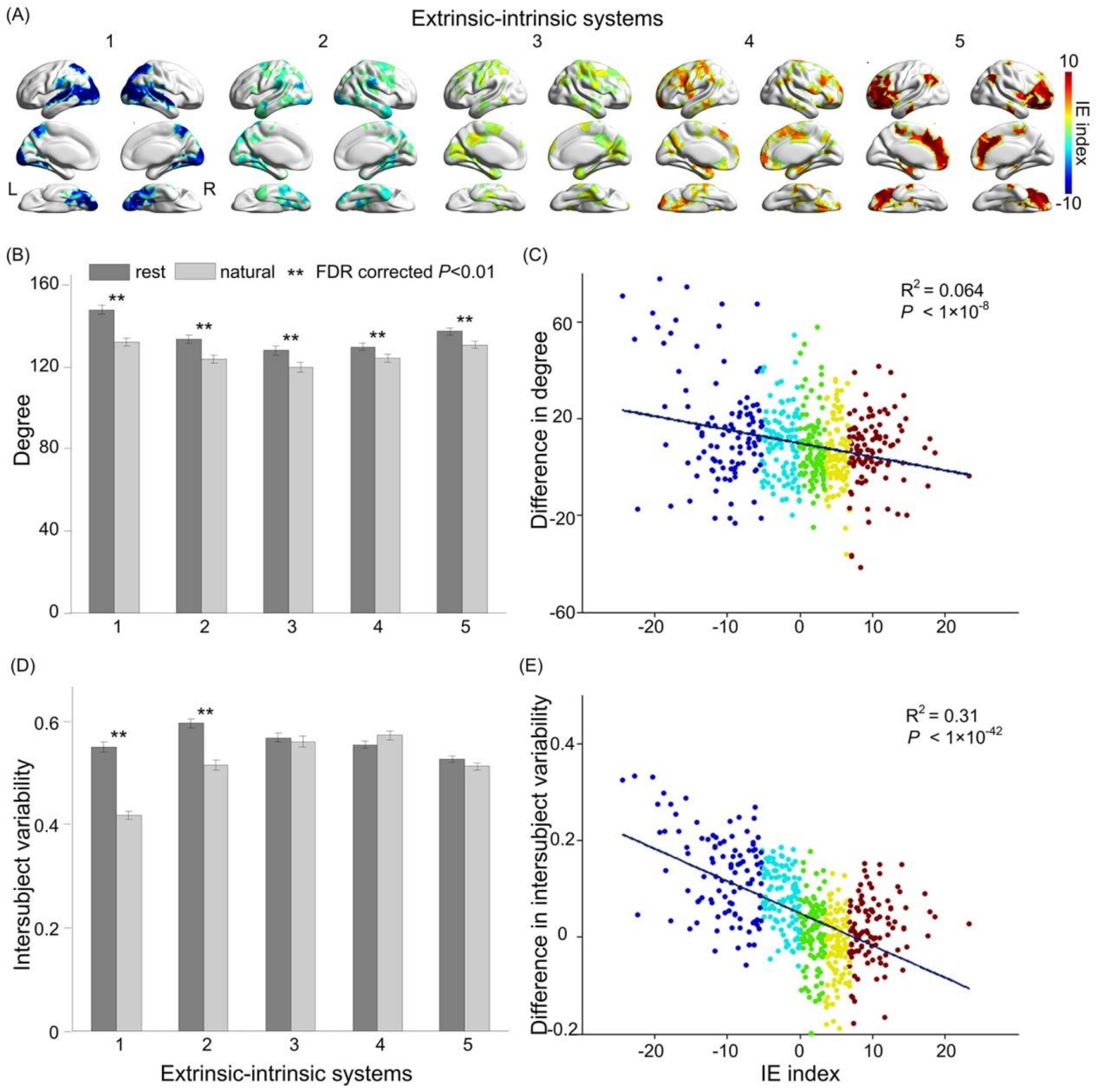


**Fig. S2 Hierarchical organization of extrinsic-intrinsic systems using 513 ROI atlas.** (A) Division of all ROIs into five extrinsic-intrinsic systems based on the IE index identified using 513 ROI atlas. Color bar signifies the IE index (warm color, intrinsic systems; cool color, extrinsic systems). (B) Average degree centrality of the five extrinsic-intrinsic systems during resting state and natural viewing (** represents significant difference between resting state and natural viewing; paired t-test, FDR-corrected $p < 0.01$). Error bars signify standard error of the mean. (C) Correlation between the IE index and changes in degree centrality between resting state and natural viewing (rest – natural). ROIs are

color-coded according to the extrinsic-intrinsic division. (D) Average inter-subject variability of the five extrinsic-intrinsic systems during resting state and natural viewing (** represents significant difference between resting state and natural viewing; paired t-test, FDR-corrected $p < 0.01$). Error bars signify standard error of the mean. (E) Correlation between the IE index and changes in inter-subject variability between resting state and natural viewing (rest – natural). ROIs are color-coded according to the extrinsic-intrinsic division.

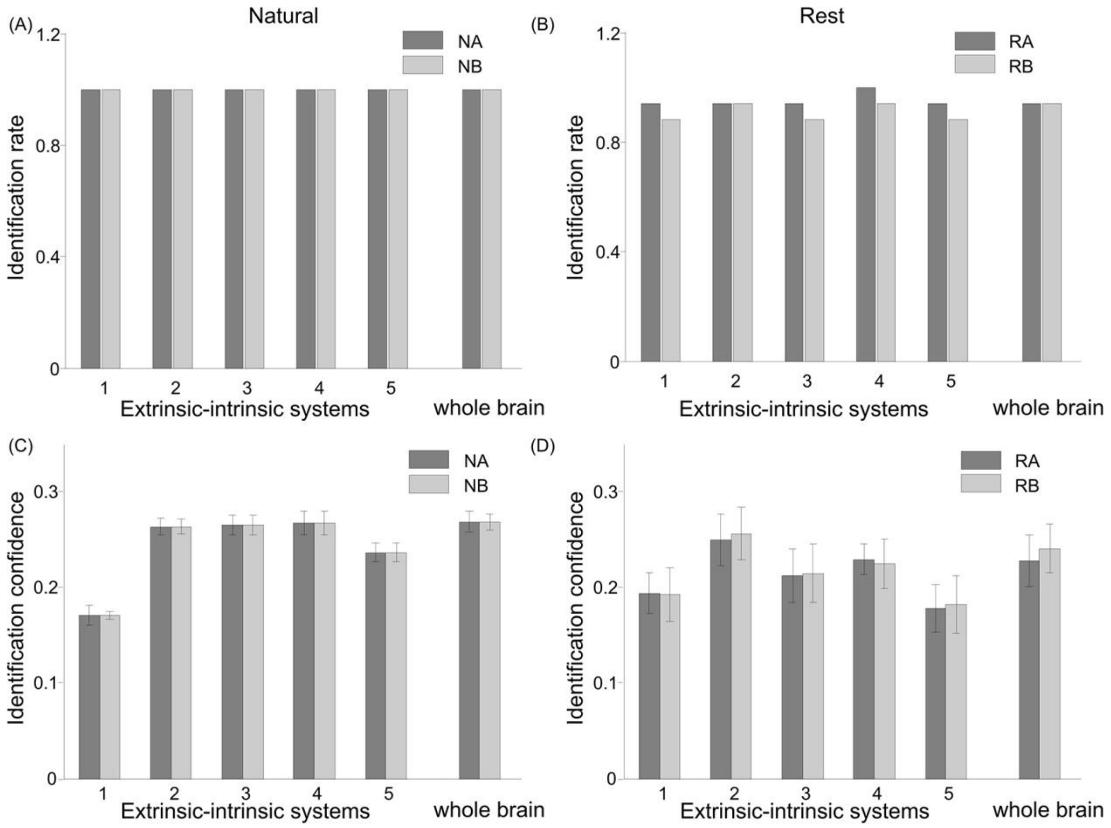


**Fig. S3 Identification rate and confidence across sessions and extrinsic-intrinsic systems using fingerprint analysis.** The extrinsic-intrinsic systems are identified using 513 ROI atlas. Bar shading (light or dark gray) indicates the session used as the target (with the other session serving as the database). NA: Natural viewing session A; NB: Natural viewing session B; RA: Resting state session A; RB: Resting state session B. Error bars signify standard error of the mean.

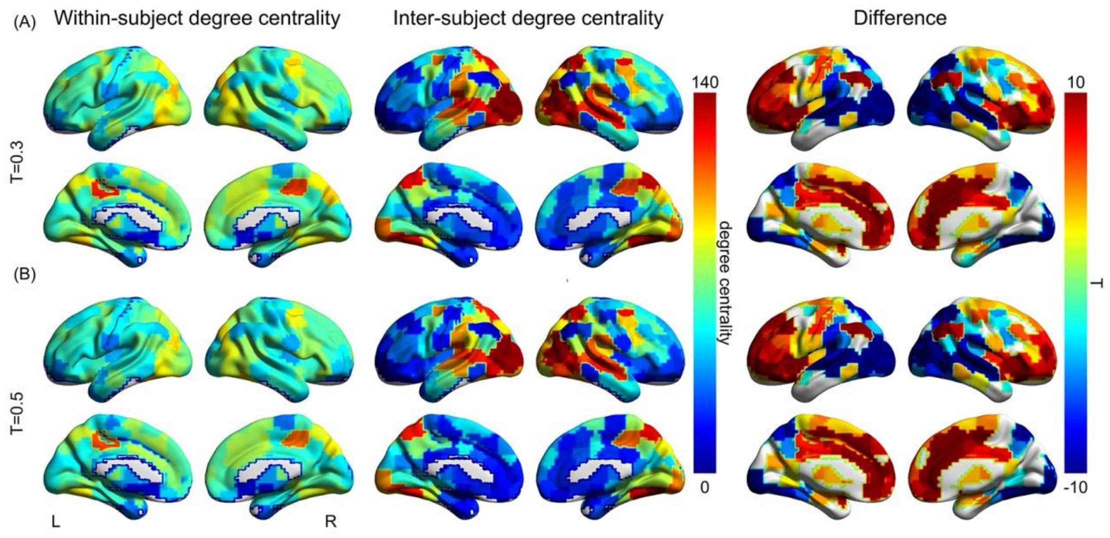


**Fig. S4 Group-level within-, inter-subject degree centrality maps, and statistical comparison between them during natural viewing using different thresholds (A) T=0.3, (B) T=0.5.** Color bar on the right of the inter-subject degree map signifies degree centrality. Color bar on the right of the difference map signifies the T statistics (warm color, within- > inter-; cool color, within- < inter; paired t-test, FDR-corrected $p < 0.05$).

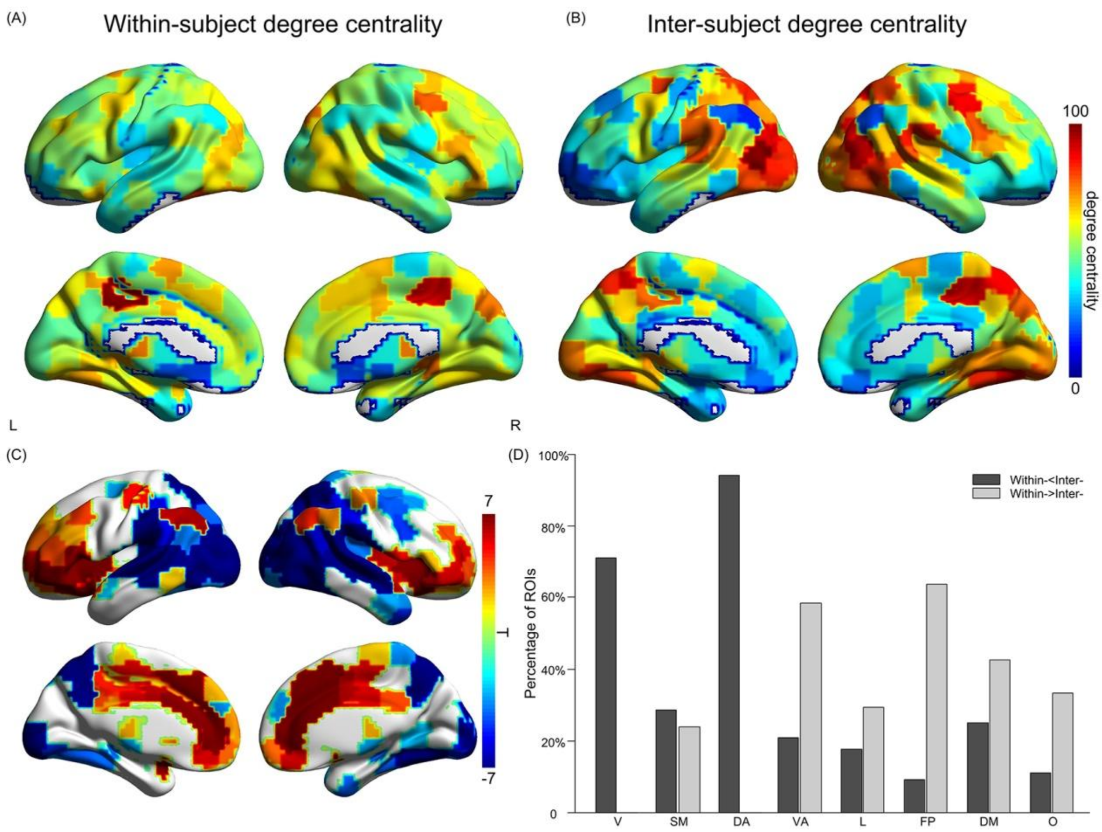


**Fig. S5 Inter-subject functional correlation analysis using sparsity threshold (T=30%).** (A) Group-level within-subject degree centrality map, (B) inter-subject degree centrality map, and (C) statistical comparison between them during natural viewing using sparsity threshold. Color bar on the upper panel signifies degree centrality. Color bar on the lower panel signifies the T statistics (warm color, within- > inter-; cool color, within- < inter; paired t-test, FDR-corrected $p < 0.05$). (D) The percentage of ROIs in each network showing significant differences between within- and inter-subject degree centrality using sparsity threshold. ROIs are sorted into the 7-network scheme(Yeo, Krienen et al. 2011). Dark gray, within- < inter-; Light gray, within- > inter-.

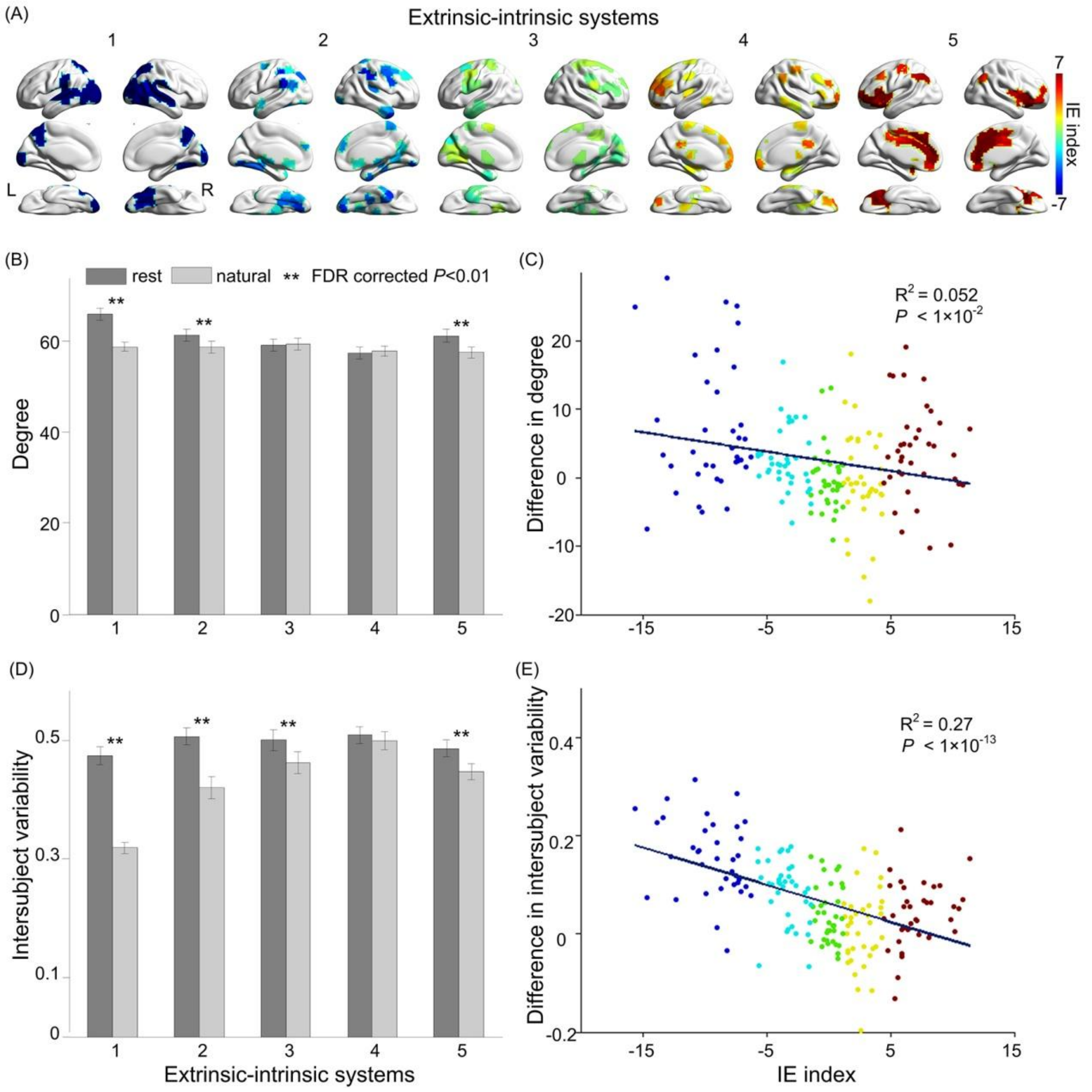


**Fig. S6 Hierarchical organization of extrinsic-intrinsic systems using sparsity threshold.** (A) Division of all ROIs into five extrinsic-intrinsic systems based on the IE index identified using sparsity threshold (T=30%). Color bar signifies the IE index (warm color, intrinsic systems; cool color, extrinsic systems). (B) Average degree centrality of the five extrinsic-intrinsic systems during resting state and natural viewing (** represents significant difference between resting state and natural viewing; paired t-test, FDR-corrected $p < 0.01$). Error bars signify standard error of the mean. (C) Correlation between the IE index and changes in degree centrality between resting state and natural viewing

(rest – natural). ROIs are color-coded according to the extrinsic-intrinsic division. (D) Average inter-subject variability of the five extrinsic-intrinsic systems during resting state and natural viewing (** represents significant difference between resting state and natural viewing; paired t-test, FDR-corrected $p < 0.01$). Error bars signify standard error of the mean. (E) Correlation between the IE index and changes in inter-subject variability between resting state and natural viewing (rest – natural). ROIs are color-coded according to the extrinsic-intrinsic division.

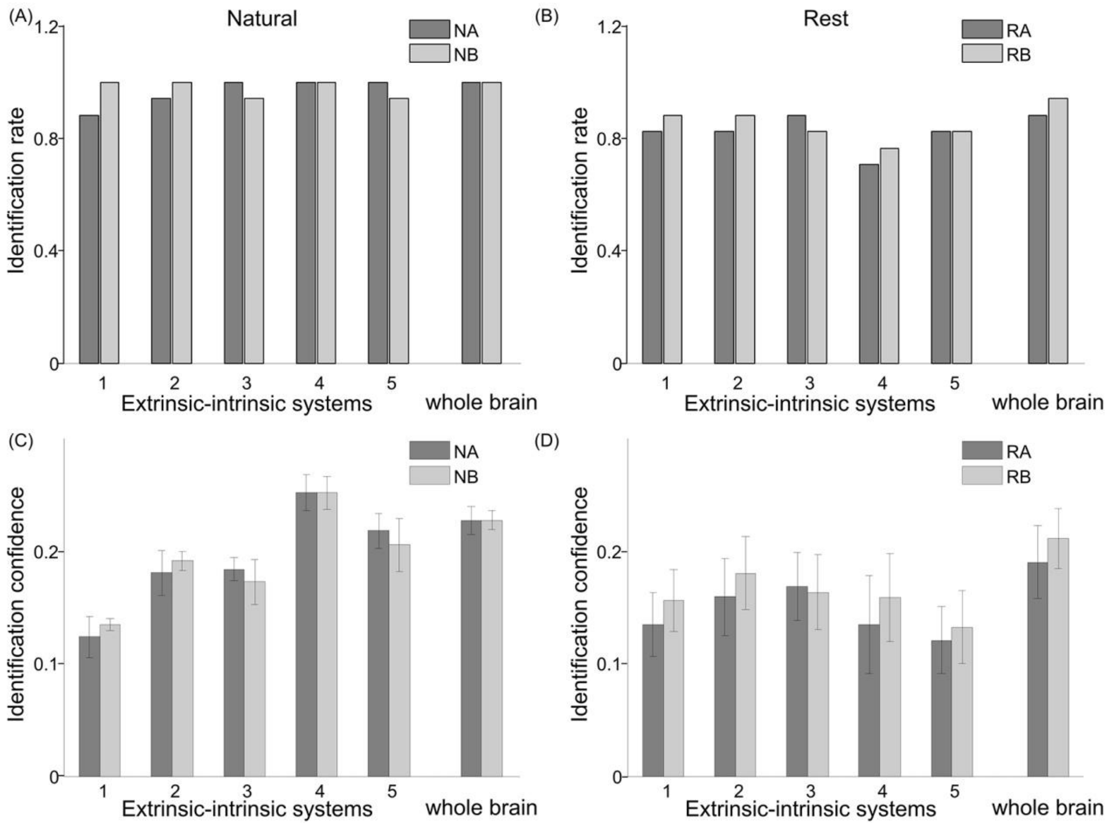


**Fig. S7 Identification rate and confidence across sessions and extrinsic-intrinsic systems using fingerprint analysis.** The extrinsic-intrinsic systems are identified using sparsity threshold (T=30%). Bar shading (light or dark gray) indicates the session used as the target (with the other session serving as the database). NA: Natural viewing session A; NB: Natural viewing session B; RA: Resting state session A; RB: Resting state session B. Error bars signify standard error of the mean.

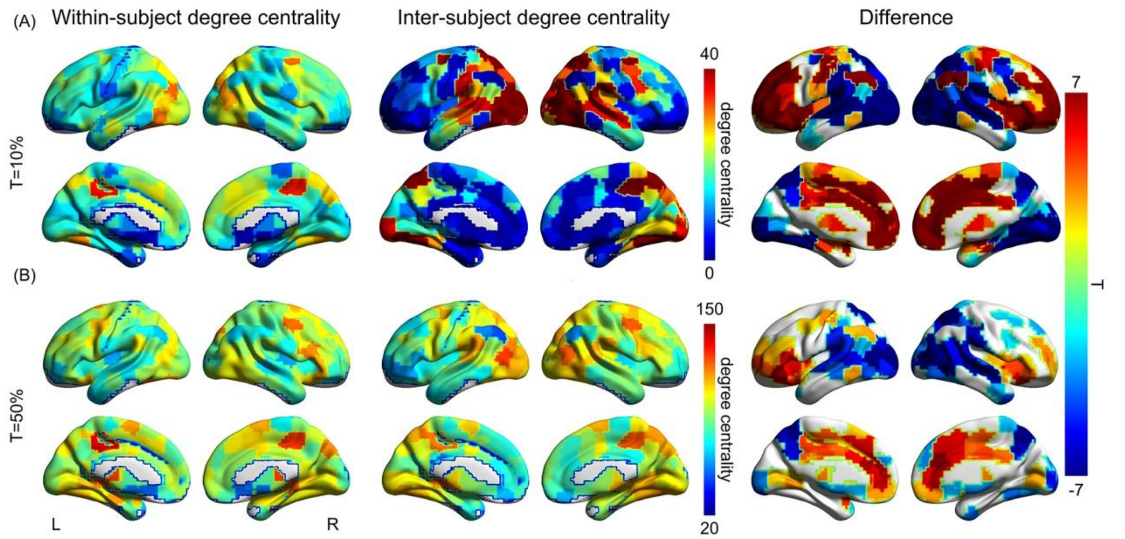


**Fig. S8 Group-level within-, inter-subject degree centrality maps, and statistical comparison between them during natural viewing using different sparsity thresholds (A) T=10%, (B) T=50%.** Color bars on the right of the inter-subject degree map signify degree centrality. Color bar on the right of the difference map signifies the T statistics (warm color, within- > inter-; cool color, within- < inter; paired t-test, FDR-corrected $p < 0.05$).

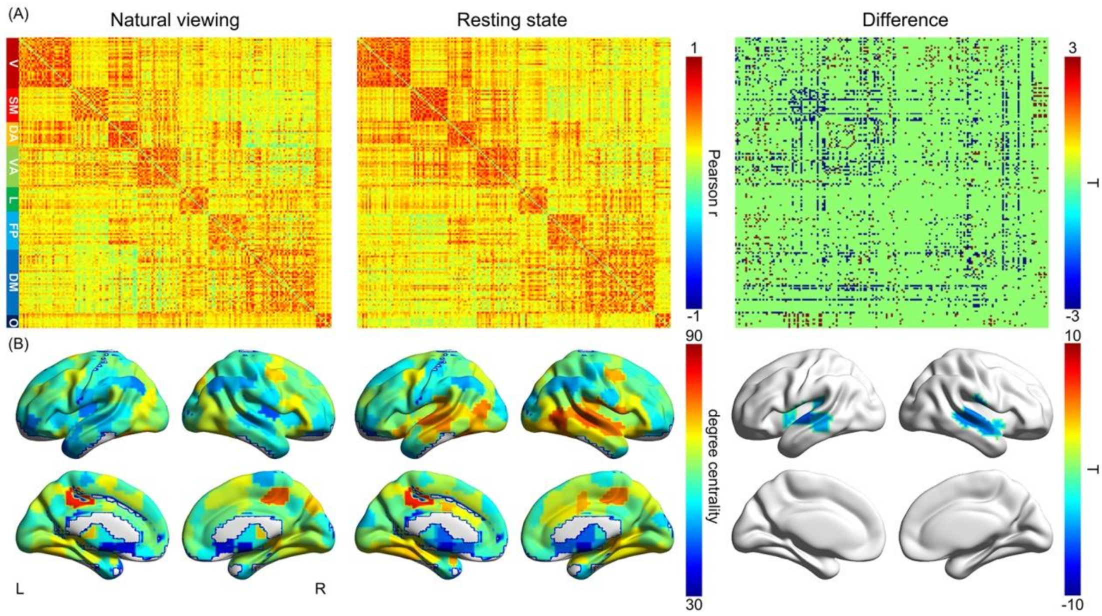


**Fig. S9 Group-level functional connectivity matrices and degree centrality maps during natural viewing and resting state.** (A) Group-level functional connectivity matrices during natural viewing (NV), resting state (RS) and statistical comparison between them. Color bar on the right of the resting state matrix signifies Pearson's correlation coefficients between each pair of ROIs. Color bar on the right of the difference matrix signifies the T statistics (warm color, NV>RS; cool color, NV<RS; paired t-test, FDR-corrected p<0.05). ROIs are organized according to the 7-network scheme (Yeo, Krienen et al. 2011), as labeled on the left of the figure (V: visual, SM: Somatomotor, DA: Dorsal attention, VA: Ventral attention, L: limbic, FP: Frontoparietal, DM: Default mode, O: Other regions). (B) group-level degree centrality maps during natural viewing, resting state and statistical comparison between them. Color bar on the right of the resting state map signifies degree centrality. Color bar on the right of the difference map signifies the T statistics (warm color, NV>RS; cool color, NV<RS;

paired t-test, FDR-corrected p<0.05).

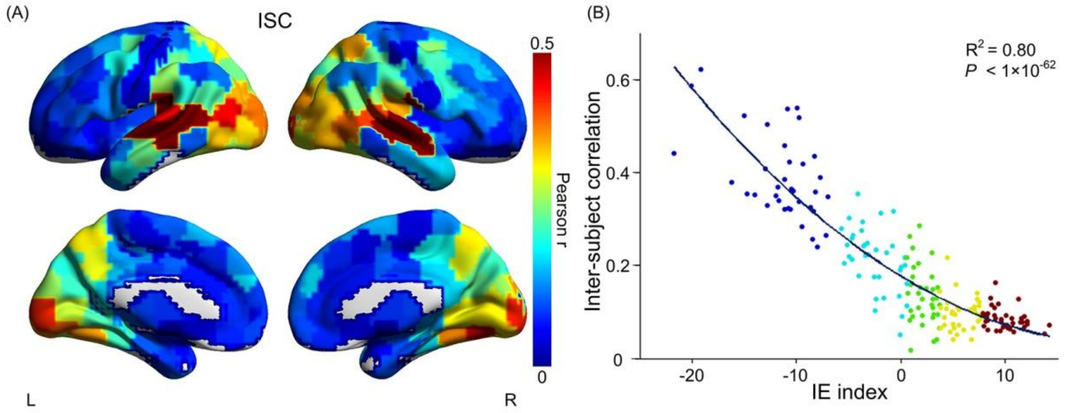


**Fig. S10 The inter-subject correlation analyses.** (A) The inter-subject correlation map during natural viewing. Color bar signifies the inter-subject correlation of each ROI. (B) Correlation between the IE index and ISC. ROIs are color-coded according to the extrinsic-intrinsic division. Dark blue line signifies a fit of exponential decay to the correlation.

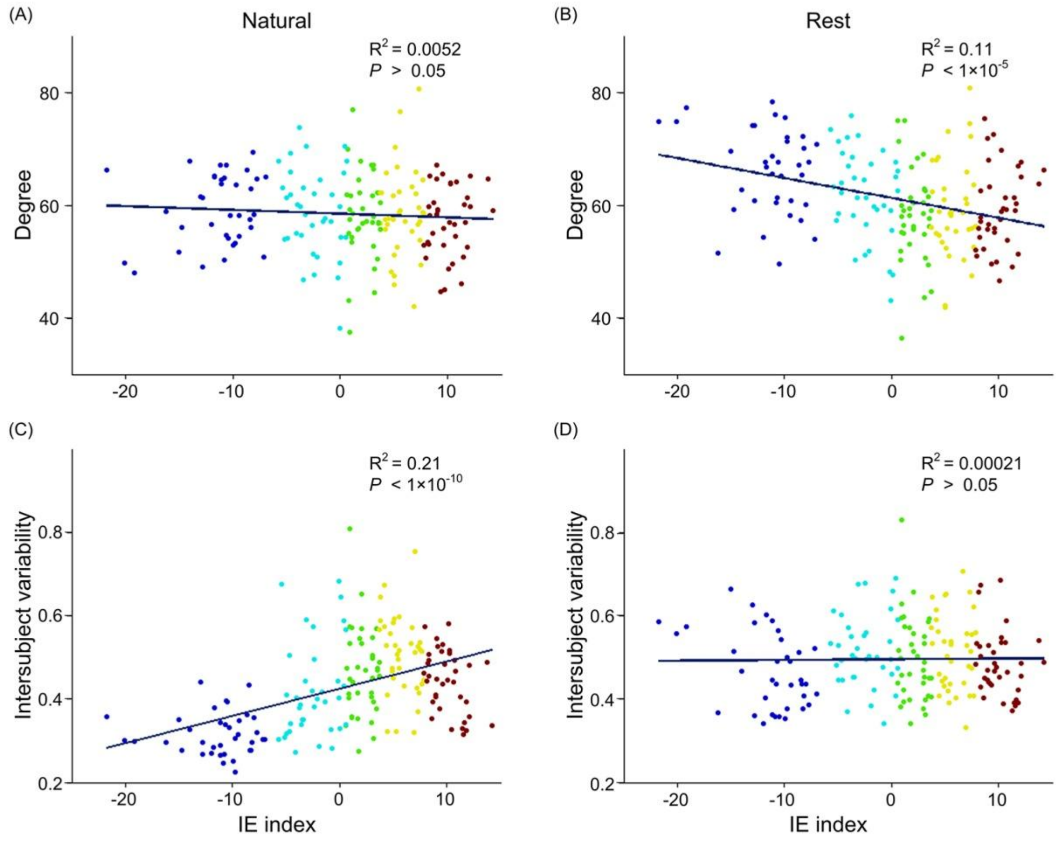


**Fig. S11 Relationship between the IE index and degree centrality.**

Correlation between the IE index and degree centrality during (A) natural viewing, (B) resting state. Correlation between the IE index and inter-subject variability during (C) natural viewing, (D) resting state. ROIs are color-coded according to the extrinsic-intrinsic division.

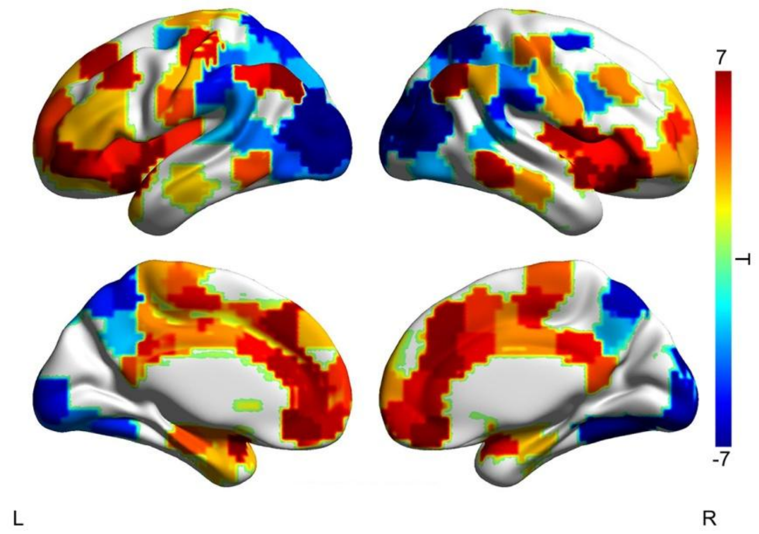


**Fig. S12 Statistical comparison between within-subject degree centrality during resting-state and inter-subject degree centrality during final 8-min segment of natural viewing.** Color bar signifies the T statistics (warm color, within- > inter-; cool color, within- < inter; paired t-test, FDR-corrected $p < 0.05$).

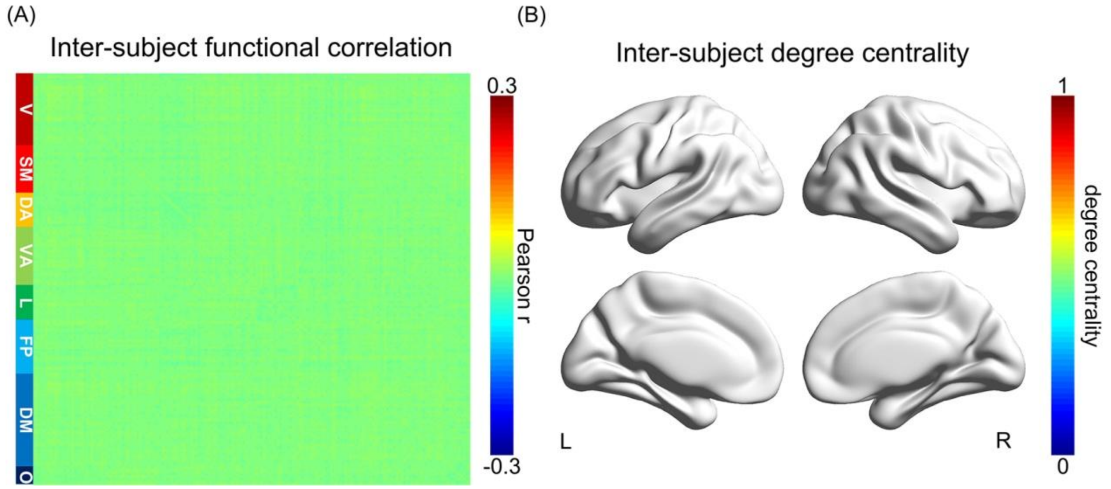


**Fig. S13 Inter-subject functional correlation matrix and degree centrality map during resting state.** (A) Group-level inter-subject functional correlation matrix during resting state. Color bar on the right of the matrix signifies Pearson's correlation coefficients between each pair of ROIs. ROIs are organized according to the 7-network scheme as labeled on the left of the figure (V: visual, SM: Somatomotor, DA: Dorsal attention, VA: Ventral attention, L: limbic, FP: Frontoparietal, DM: Default mode, O: Other regions). (B) group-level inter-subject degree centrality maps during resting state. Color bar on the right of the resting state map signifies degree centrality.
